# The Identification of *Fritillaria* Species Using Hyperspectral Imaging with Enhanced One-Dimensional Convolutional Neural Networks via Attention Mechanism

**DOI:** 10.3390/foods12224153

**Published:** 2023-11-16

**Authors:** Huiqiang Hu, Zhenyu Xu, Yunpeng Wei, Tingting Wang, Yuping Zhao, Huaxing Xu, Xiaobo Mao, Luqi Huang

**Affiliations:** 1 School of Electrical and Information Engineering, Zhengzhou University, Zhengzhou 450001, China; 2 China Academy of Chinese Medical Sciences, Beijing 100070, China

**Keywords:** medicinal and edible plants, *Fritillaria* identification, hyperspectral imaging, one-dimensional convolutional neural network, attention mechanism

## Abstract

Combining deep learning and hyperspectral imaging (HSI) has proven to be an effective approach in the quality control of medicinal and edible plants. Nonetheless, hyperspectral data contains redundant information and highly correlated characteristic bands, which can adversely impact sample identification. To address this issue, we proposed an enhanced one-dimensional convolutional neural network (1DCNN) with an attention mechanism. Given an intermediate feature map, two attention modules are constructed along two separate dimensions, channel and spectral, and then combined to enhance relevant features and to suppress irrelevant ones. Validated by *Fritillaria* datasets, the results demonstrate that an attention-enhanced 1DCNN model outperforms several machine learning algorithms and shows consistent improvements over a vanilla 1DCNN. Notably under VNIR and SWIR lenses, the model obtained 98.97% and 99.35% for binary classification between Fritillariae Cirrhosae Bulbus (FCB) and other non-FCB species, respectively. Additionally, it still achieved an extraordinary accuracy of 97.64% and 98.39% for eight-category classification among *Fritillaria* species. This study demonstrated the application of HSI with artificial intelligence can serve as a reliable, efficient, and non-destructive quality control method for authenticating *Fritillaria* species. Moreover, our findings also illustrated the great potential of the attention mechanism in enhancing the performance of the vanilla 1DCNN method, providing reference for other HSI-related quality controls of plants with medicinal and edible uses.

## 1. Introduction

Plants possessing both medicinal and edible properties, typically known as “medicinal and edible plants”, have been providing nourishment and healing to humanity for centuries [1,2]. These plants embody the philosophy of “food as medicine and medicine as food”, offering unique benefits and playing a significant role in daily life and well-being. Due to their dual uses and benefits, medicinal and edible plants have gained increasing popularity and widespread acceptance among the general public [3,4]. They provide a wealth of nutritional, sensory, and bioactive compounds that deliver both prophylactic and therapeutic effects [5,6] and have been traditionally used in folk medicines and cuisines across cultures. In the modern world, the trend towards preventive healthcare and natural remedies has made medicinal and edible plants an attractive alternative for promoting health, preventing disease, and improving the quality of life [7,8]. However, the quality of these plants can vary significantly depending on factors such as growing environment, harvesting, and processing methods. Therefore, it is crucial to implement comprehensive quality control measures to ensure their safety, efficacy, and overall quality as both food and medicine.

Being one of the largest genera in the family Liliaceae, *Fritillaria* boasts a wide variety of species and rich resources [9], primarily found in the temperate and tropical regions of the Northern Hemisphere, particularly along the North Coast of the Mediterranean Sea, as well as in Iran, Turkey, and other regions [10,11]. In China, it is often encountered as Beimu and is a well-known plant with both medicinal and edible uses. *Fritillaria* has been used for a prolonged period in traditional Chinese medicine for its respiratory and cardiovascular benefits, thanks to its steroidal compounds [12]. It is also used as a food ingredient in many Chinese dishes due to its distinctive flavor and texture [11]. Moreover, *Fritillaria* is widely used in the daily chemical industry [13,14].

Due to the diverse and intricate origins of medicinal and edible plants belonging to the genus *Fritillaria* [15], the latest version of the Chinese Pharmacopoeia has included six types of Beimu medicinal materials, namely Fritillariae Cirrhosae Bulbus, Fritillariae Thunbergii Bulbus, Fritillariae Hupehensis Bulbus, Fritillariae Ussuriensis Bulbus, Fritillariae Pallidiflorae Bulbus, and Bolbostemmatis Rhizoma (Tubeimu). The medicinal and nutritional values of various *Fritillaria* species differ significantly [16]. Among them, *Fritillaria cirrhosa* Bulbus (FCB) is the most valuable, potent, and sought-after herbal material [17,18]. However, the best quality FCB primarily originates from wild resources located in high-altitude and cold regions, resulting in a significantly higher market price compared to other *Fritillaria* species, ranging from 10 to 200 times higher [19,20]. Additionally, unscrupulous traders may replace or contaminate valuable herbs with cheaper varieties to maximize their profits due to this price disparity [16]. Given the close genetic relationship and morphological similarity among all species of *Fritillaria*, relying solely on appearance makes it exceedingly challenging to accurately identify them. Therefore, there is an urgent need to develop practical technological approaches that can effectively differentiate between various *Fritillaria* herbal species and their adulterants. Such identification techniques are crucial for ensuring the authenticity, safety, and efficacy of *Fritillaria*-based products. Specifically, the rapid and precise identification of authentic *Fritillaria* medicinal materials remains a significant challenge that needs to be addressed.

For the related quality control of medicinal and edible plants, commonly employed detection methods include chemical experiments, such as alkaloid content [21], as well as DNA barcoding [19,22] and MS-based metabolomic methods [23], among others [17,24,25]. In practice, these above-mentioned methods can be employed to identify various *Fritillaria* species by determining the relative content of their primary chemical components. However, there are still many limitations. For example, chemical testing usually damages samples and requires personnel with corresponding professional knowledge; additionally, the experiment itself takes a long time. Moreover, these methods involve selective examinations that can only assess a limited number of samples [26,27,28]. Therefore, it is not suitable for mass testing and online quality measurement, especially for market supervision. Consequently, a fast, non-destructive, and efficient method is urgently needed to identify and classify *Fritillaria* from different sources.

In recent decades, extensive research has propelled the field of image analysis forward, yielding a diverse array of innovative methods and cutting-edge devices [29]. Among the notable advancements in this domain, hyperspectral imaging stands out, distinguished by its manifold advantages. This technology excels in its capacity to capture data across a broad spectrum of wavelengths, bestowing a meticulously detailed spectral signature upon each pixel in an image [30]. The utility of hyperspectral imaging encompasses a spectrum of benefits: it enables enhanced spectral discrimination, fostering improved classification and detection, while also facilitating non-invasive analysis. The versatility of hyperspectral imaging has led to its pervasive adoption across an array of disciplines. These encompass medical diagnostics [31], agriculture [32], environmental science [33], and more. What sets hyperspectral imaging apart is its unique proficiency in extracting intricate spectral data from images, thus considerably amplifying the scope for analysis and exploration within these fields [34].

Recently, hyperspectral imaging (HSI) technology has emerged as a promising tool for online and non-destructive quality control of herbs with medicinal and edible values, offering a unique advantage in the reflecting of the comprehensive chemical composition information of tested samples [35,36]. In particular, by integrating spectroscopic and imaging techniques, HSI enables the simultaneous acquisition of information on multiband spectral variables and the spatial location of detected objects, thereby increasing the amount of information obtained [37,38,39]. Coupled with chemometrics, there are also relevant studies on the quality control of medicinal and edible plants. For instance, Ru et al. [40] utilized visible and near-infrared (VNIR) and shortwave infrared (SWIR) spectral ranges to create a spectrum-image fusion (VNIR-SWIR-FuSI). They then employed machine learning algorithms to classify the geographical origins of Rhizoma Atractylodis Macrocephalae (RAM). The results demonstrated that HSI combined with VNIR-SWIR-FuSI is an effective method for determining the geographical origin of RAM, achieving an impressive accuracy of 97.3% and 93.2%. Han et al. [41] developed a combination of spectral analysis and image processing to classify *Glycyrrhiza* seeds, showing that HSI technology is a useful tool for the rapid and non-destructive classification of seeds. To identify the origin of *Lycium barbarum*, Wang et al. [42] selected the most informative wavelengths using one-way analysis of variance (ANOVA); then, he used machine learning algorithms to classify HSI data based on their origin. The proposed model achieved the highest accuracy of 95.25%. Furthermore, Yao et al. [43] proposed a non-destructive detection method based on HSI technology, CARS-PCA, and MPA-LSSVM for classifying different grades of *Panax notoginseng* powder based on their chemical composition, demonstrating a promising method with an accuracy of 95%.

Except for some necessary preprocessing processes, the above-mentioned traditional methods mainly include two processes: spectral wavelength selection and classification model construction. Within a large number of spectral bands, selecting an appropriate wavelength can directly influence the subsequent model classification performance [44,45,46]. Thus, it usually requires domain knowledge and is usually a trial-and-error procedure. In the past few years, deep learning, especially the use of the convolutional neural network (CNN) as a fused feature extraction and classification method [47,48,49], is known to deal well with high dimensionality, and it has achieved remarkable results for different applications [50,51,52,53].

Nowadays, successful studies have been conducted using HSI techniques and deep learning for the purpose of quality control of medicinal and edible plants. For example, Xiao et al. [54] conducted a study on geographical origin identification of Radix Astragali using visible/short-wave near-infrared and near-infrared HSI. They proposed a CNN-based feature extraction and data fusion approach, achieving high classification accuracy for different regions of origin, with all of the models obtaining over 98% accuracy. Similarly, Li et al. [55] introduced an accurate method for predicting the soluble solid content of dried Hami jujube using SWIR hyperspectral imaging. Their research emphasizes the promising application of hyperspectral imaging and CNN in quality prediction within the food industry, particularly in the quality control of dried fruits. Another successful deep learning model was developed by He et al. [56], who presented a novel approach for the simultaneous determination of five micro-components in *Chrysanthemum morifolium* (Hangbaiju) using near-infrared hyperspectral imaging coupled with convolutional neural networks (CNN) with a wavelength selection. The study findings demonstrate the accuracy and efficiency of this method for the quality control of Hangbaiju. In their study, Zhang et al. [57] utilized near-infrared HSI to determine chemical compositions in dry black goji berries and proposed a deep learning-based regression approach. The approach accurately predicted the concentrations of various chemical components present in the berries. These studies collectively demonstrate the potential of deep learning methods in improving HSI analysis for various applications in medicinal and edible plants.

Motivated by successful applications of HSI in the identification and prediction of medicinal and edible plants, this study aimed to explore the feasibility of employing HSI for the authentication of *Fritillaria* species. In above-mentioned studies [54,55,56,57], experimental results showed the effectiveness and potential of the 1DCNN method proposed. Notwithstanding, the HSI data acquired are characterized by high dimensionality, collinearity, and redundancy. Currently, a deficiency of these 1DCNN models [54,55,56,57] is that they do not explicitly consider the relevance and redundancy of raw spectral data, which limits their performance improvement. To address these challenges, we explore 1DCNN improvement with three proposed attention mechanism modules [58]. By virtue of the attention mechanism, the network can dynamically enhance discriminative features while suppressing irrelevant ones. The contributions of our study are summarized as follows:

- We propose three feature optimization modules for a 1DCNN: the channel attention module (CAM), the spectral attention module (SAM), and the joint channel–spectral attention module (CSAM). The CAM enhances classification-relevant spectral features and suppresses irrelevant ones by modeling the interdependence between convolution feature channels. The SAM selectively attends to informative spectral features while ignoring uninformative ones. The CSAM combines channel and spectral attention mechanisms to optimize feature mapping and fuse the output of the two modules. With the help of these attention modules, 1DCNNs can effectively select informative spectral bands and generate optimized features.
- The 1DCNN network that uses the proposed attention mechanism is explored as an end-to-end approach to the identification of *Fritillaria*. To the best of our knowledge, this is the first time that an attention-based 1DCNN has been applied to the identification of *Fritillaria*. With the data collected on *Fritillaria*, the CSAM–1DCNN maintained remarkable classification accuracies of 98.97% and 99.35% under both VNIR and SWIR lenses, respectively, for binary classification between Fritillariae Cirrhosae Bulbus (FCB) and other non-FCB species. Additionally, for eight-category classification among *Fritillaria* species, it still achieved a high level of precision, with an extraordinary accuracy of 97.64% and 98.39%, respectively.
- Our findings illustrated the great potential of the attention mechanism in enhancing the performance of the vanilla 1DCNN method. Nowadays, research on the application of the attention mechanism in the analysis of medicinal and edible plants using hyperspectral imaging remains limited. Consequently, our study provides new references for other HSI-related quality controls of herbal medicines, expecting to further improve its performance.

This reminder of this paper is organized as follows: In Section 2, we present sample data collection involving necessary pre-processing techniques, and the basics of a 1D convolutional neural network, as well as the attention mechanism proposed, are described. The results are presented, and a comparative analysis is carried out with discussions in Section 3, and in Section 4, the article is concluded.

## 2. Materials and Methods

### 2.1. Samples Preparation

This study examined six varieties of Beimu, including Fritillariae Cirrhosae Bulbus (FCB) and non-FCB. The non-FCB group consisted of five species, namely Fritillariae Thunbergii Bulbus (FTB), Fritillariae Hupehensis Bulbus (FHB), Fritillariae Ussuriensis Bulbus (FUB), Fritillariae Pallidiflorae Bulbus (FPB), and Bolbostemmatis Rhizoma (BSR). Additionally, the FCB group comprised three commodity specifications: QingBei (QB), SongBei (SB), and LuBei (LB). All of the *Fritillaria* samples used in this study were verified by professors and met the standards outlined in the Pharmacopoeia of the People’s Republic of China. The samples were supplied by the China Academy of Chinese Medical Science and stored in vacuum prior to experimentation.

### 2.2. Hyperspectral Imaging System Acquisition

**Figure 1:**
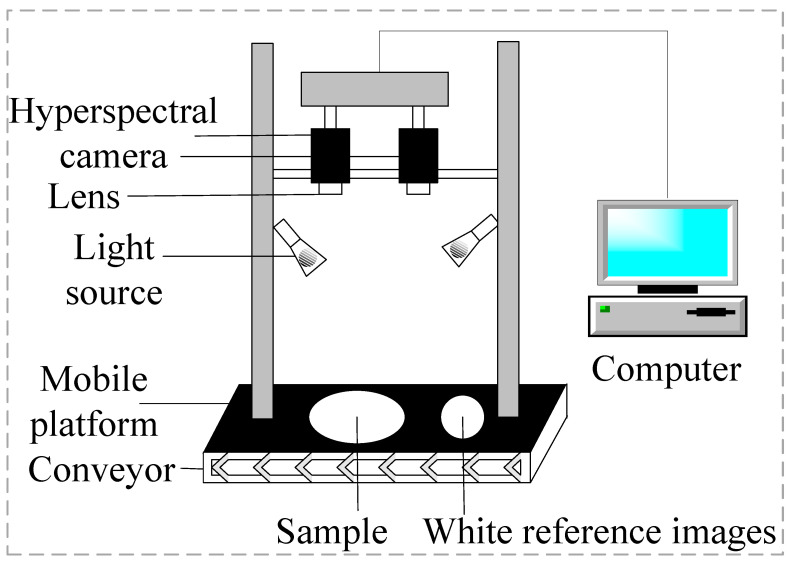
Hyperspectral imaging acquisition system.

The hyperspectral imaging system utilized in this study is the HySpex series of hyperspectral imaging spectrometers, manufactured by Norsk Elektro Optikk AS (Oslo, Norway), as illustrated in Figure 1. This system comprises two halogen lamps, a CCD detector, and two lenses, namely, the SN0605 VNIR (VNIR) lens and the N3124 SWIR (SWIR) lens. The VNIR lens covers a spectral range spanning from 410.42 to 990.10 nm, consisting of a total of 108 distinct bands. On the other hand, the SWIR lens encompasses the spectral range of 948.72 to 2512.97 nm, featuring a more extensive range of 288 bands. For operation, the mobile platform is equipped with an integrated computer and software. The distance between the hyperspectral imager’s lens and the samples is meticulously set at a range of 20–30 cm. The platform moves at a steady speed of 1.5 mm/s during data acquisition. The integration time for the VNIR and SWIR lenses is precisely set at 9000 µs and 4500 µs, respectively.

### 2.3. Data Preprocessing

During acquisition, the non-uniformity of light intensity and the interference of dark current can produce uneven output images [59], which hampers subsequent data analysis. Consequently, it is essential to calculate relative reflectance using a dark and white reference image. The corresponding correction method is calculated as the following equation:

(1) $$I_{new}=\frac{I_{raw}-I_{dark}}{I_{white}-I_{dark}}$$

where *I_new_* denotes the corrected image, *I_raw_* is the original hyperspectral image, *I_dark_* represents the dark reference images, and *I_white_* represents the white reference images.

Following the correction of hyperspectral images, a sampling method based on grids was devised to acquire uniform samples from each hyperspectral image of the various *Fritillaria* species, within the band ranges of 410.42–990.10 nm and 948.72–2512.97 nm, which consisted of 396 bands. Each sample was obtained by calculating the average reflectance within the corresponding region of interest (ROI), and the details of *Fritillaria* spectra dataset are presented in Table 1.

Aiming to eliminate the impact of surface scattering and path transformation on diffuse reflection [60], we adopted the standard normal variate (SNV) to preprocess HSI data. Mathematically, the SNV can be defined as follows:

(2) $$X_{SNV}=\frac{x-\bar{x}}{\sigma }$$

where x, $\bar{x}\;$, and σ represent samples, the mean, and the standard deviation of the sample, respectively.

### 2.4. Basic Architecture of a 1DCNN Model

Convolutional neural networks (CNNs) typically comprise five key components: an input layer, one or more convolutional layers, one or more pooling layers, one or more fully connected layers, and an output layer. Initially proposed by LeCun et al. [61], CNNs are specialized networks for applications, mainly images. For one-dimensional spectral data, the convolution layer and the pooling layer are mainly adjusted in a 1DCNN. The main components of a 1DCNN and its work principle during the training process are described in the following sections.

**One-dimensional convolution layer:** The convolutional layer is considered the most essential component of a 1DCNN model. This layer uses a set of learnable filters to convolve the input data, enabling it to effectively extract local features. As the filter slides along the one-dimensional input space, it performs dot product operations at each position, producing a set of output features, or feature maps. Typically, this layer also includes bias terms and activation functions to introduce non-linear properties. The calculation of one-dimensional convolution can be presented as follows:

(3) $$Y_{l}^{j}=\delta \left(\sum_{i=\left[1,M\right]}\left(X_{l-1}^{i}*W_{l}^{ij}\right)+b_{l}^{j}\right)$$

where $Y_{l}^{j}$ represents the feature mapping of the *j*th output in the *l*th layer, while $X_{l-1}^{i}$ denotes the *i*th input feature map of the (*l* − 1)th layer. The weight value of the convolution kernel in the *i*th channel of the *j*th convolution kernel in the *l*th layer can be represented by $W_{l}^{ij}$, and $b_{l}^{j}$ denotes the bias vector of the *j*th convolution kernel in the *l*th layer. The convolution operation is represented by {*}, with the number of channels in the convolution kernel denoted by *M*. The activation function is denoted by $\delta \left(x\right)$.

**One-dimensional pooling layer:** The pooling layer is a crucial element in a 1DCNN, as it plays a vital role in reducing the dimensionality and number of output features. During this process, input feature maps are divided into non-overlapping regions, and the values within each region are compressed into a single output value, effectively reducing the spatial size of the feature maps. The most commonly used methods for pooling layers are max and average pooling. Different pooling operations can be implemented depending on the specific requirements of the model. Max pooling extracts the maximum value within each region, while average pooling computes the average value of all the elements in the region. By reducing the computational cost of the network and simultaneously enhancing its performance, the pooling layer becomes an indispensable component of a 1DCNN.

**Fully connected layer:** The fully connected layer is responsible for connecting the output of the previous layer to the final output layer. In this layer, each neuron is connected to all the neurons in the previous layer, and the weights between the neurons are learned during training. The fully connected layer is responsible for performing the classification or regression task and producing the final output of the 1DCNN. The output of each neuron is determined as follows:

(4) $$G_{l}^{i}=\delta \left(\left(w_{l}^{i}*x_{l-1}\right)+b_{l}^{i}\right)$$

where $G_{l}^{i}$ represents the output of the *i*th neuron in layer *l*, while $x_{l-1}$ denotes the output values of all neurons at layer *l* − 1. The weight value and bias vector of the *i*th neuron in the *l*th layer are denoted by $w_{l}^{i}$ and $b_{l}^{i}$, respectively, and $\delta \left(x\right)$ represents the activation function.

**SoftMax layer:** The SoftMax layer is commonly used as the final output layer in classification tasks. It transforms the output of the previous layer into a probability distribution over the possible classes, allowing the model to predict the probability of each class. To achieve this, the SoftMax function takes the exponential of each output value and normalizes them, ensuring that they sum up to 1. This normalization step is critical for obtaining accurate class probabilities and is essential for achieving high accuracy in classification tasks. The principle of the SoftMax layer can be computed as follows:

(5) $$P\left(V_{i}\right)=\frac{e^{V_{i}}}{\sum_{K=1}^{C}e^{V_{K}}}$$

In the equation above, $P\left(V_{i}\right)$ represents the probability of the output of the *i*th category of the output neuron, while $e^{V_{i}}$ denotes the corresponding nonlinear prediction probability of the *i*th class. Here, C represents the number of output nodes, and $V_{i}$ is the activation value of the *i*th node.

### 2.5. Attention Mechanism

#### 2.5.1. Channel Attention Module

After the convolution operation, different convolution kernels obtain different feature maps, which contribute to the final contribution in different degrees; therefore, some features may not be related. The CAM technique is designed to selectively enhance relevant features while suppressing irrelevant ones, achieved by explicitly modeling the interdependence between convolution feature channels, leading to better discrimination.

Assuming that the original input features are denoted by $Y=\left[y_{1},y_{2},\ldots y_{x}\right]$, CAM compresses the input features of each channel into a one-dimensional channel statistical vector Z, ($Z\in R^{x\times 1})$ by using the global average and max pooling layer. The method for calculating the *i*th element of *Z* is given as follows:

(6) $$Z_{i}^{Avg}=\frac{1}{L}\sum_{j=1}^{L}y_{i}\left(j\right)$$

(7) $$Z_{i}^{max}=\max\left\{y_{i}\left(j\right)\right\}$$

where $y_{i}$ is a one-dimensional input feature with a length of *L*.

The channel information vector $Z^{*}$ is obtained by applying one-dimensional convolution and nonlinear layers, with a convolution kernel size of 1 × 1. The computation process for obtaining $Z^{*}$ is specified in this equation:

(8) $$Z^{*}=\omega \left(f_{2}\left(\varphi \left(f_{1}\left(Z_{i}^{max}\right)\right)\right)\right)+\omega \left(f_{2}\left(\varphi \left(f_{1}\left(Z_{i}^{Avg}\right)\right)\right)\right)$$

where $f_{1}$ and $f_{2}$ are convolution layers with a convolution kernel size of 1 × 1. $Z_{i}^{*}$ refers to the weight of the *i*th channel. φ and ω denote the ReLU and Sigmoid functions, respectively.

To prevent network degradation during the training process, a residual structure is integrated into the channel attention mechanism. The computation for obtaining the final output CAM is as follows:

(9) $$Y_{CAM}=Y+Y\cdot Z^{*}$$

where $Y=\left[y_{1},y_{2},\ldots y_{x}\right]$ and $Z^{*}=[Z_{1}^{*},Z_{2}^{*},Z_{3}^{*},\ldots Z_{x}^{*}]$, representing original spectral features and channel information, respectively.

#### 2.5.2. Spectral Attention Module

Spectral wavelengths in hyperspectral images are known for their high degree of dimensionality, as well as their collinearity and redundancy. In other words, the final contribution of different wavelength to the final classification is not equal, and some wavelength bands may even bring interference. The Spectral Attention Module (SAM) is dedicated to enhancing classification-related spectral features and to ignoring irrelevant spectral features. This allows the SAM to locate and optimize the convolutional neural network’s response to the relevant spectral bands, thus improving the efficiency of the network.

To be specific, let *Y* denote the original input features, such that $Y=\left[y_{1},y_{2},\ldots y_{z}\right]$, where $y_{j}\in R^{x\times 1}$ represents the feature information of the *j*th spectral band. The calculation method for obtaining the local information feature vector, denoted as $T^{*}$, is outlined below:

(10) $$T^{*}=\varphi \left(f\left(y_{j}\right)\right)$$

where f represents the x channels, and a 1 × 1 convolution kernel with a single convolution kernel is employed, while φ is a ReLU activation function. The residual structure is also incorporated in a similar manner as the CAM, and the final output can be expressed as such:

(11) $$Y_{SAM}={Y+T}^{*}\cdot Y$$

#### 2.5.3. Joint Channel and Spectral Attention Module 

The CSAM combines the strengths of both CAM and SAM to optimize the input feature Y. By leveraging the advantages of these two methods, CSAM further improves the feature learning ability and encourages the network to learn more discriminant features. That is, the CAM and the SAM work together to enhance the channel and spectral features from different perspectives in turn, as represented in this equation:

(12) $$Y_{CSAM}=F_{CAM}\left(F_{SAM}\left(Y\right)\right)$$

### 2.6. Proposed CSAM–1DCNN

**Figure 2:**
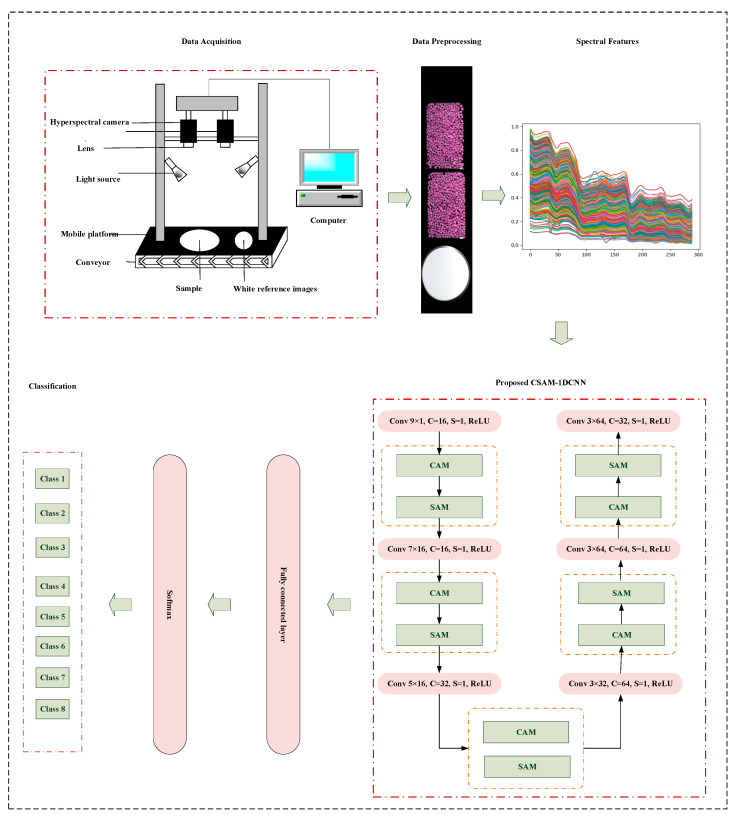
The workflow employed for the classification of the *Fritillaria* species.

Building upon the previously discussed attention modules, we have devised a multi-attention 1DCNN framework tailored for the authentication of *Fritillaria* species. This network architecture comprises six one-dimensional convolutional modules, integrated with five CSAMs, culminating in a fully connected layer and a SoftMax layer. The incorporation of CSAMs behind each convolutional module serves the dual purpose of enhancing feature optimization and facilitating the selection of pertinent spectral bands. Figure 2 delineates the workflow employed for the classification of the *Fritillaria* species.

Table 2 lists the parameters for CSAM–1DCNN when analyzing spectral data under the SWIR lens. The CSAM facilitates the selective learning of spectral information, and during the training process, a relatively small convolution kernel is selected for the first level of convolution. We chose a max-pooling layer with a pooling stride of 1 to aggregate spectral characteristics and eventually compressed the feature length to 4 × 1. In the later convolution process, we reduced the number of feature maps as was appropriate to avoid redundancy, enhance the efficiency of feature extraction, and improve the training speed of the fully connected network. To demonstrate the universality of the proposed model, we employed an identical convolutional network structure under VNIR and SWIR lenses except for the first layer input for different bands.

## 3. Results and Discussion

In this section, the performance evaluation metrics, software tools, and configurations used are first presented. Then, two experiments, including binary classification and a more complex eight-classification, were conducted. Each experiment consists of two stages: (1) performance comparison between conventional algorithms and the vanilla 1DCNN model and (2) the effect of different attention mechanism modules on the 1DCNN performance.

### 3.1. Experimental Settings

**Performance Evaluation Metrics:** Four performance indices, including accuracy, precision, sensitivity, and confusion matrix, are adopted to indicate the classification effectiveness of the model, which are denoted as follows, respectively:

(13) $$Accuracy=\frac{TP+TN}{TP+FP+TN+FN}$$

(14) $$Precision=\frac{TP}{TP+FP}$$

(15) $$Sensitivity=\frac{TP}{TP+FN}$$

where TP, TN, FP, and FN refer to true positive samples, true negatives samples, false positives, and false negatives samples, respectively. Accuracy, precision, and sensitivity are expressed as percentages, ranging from 0 to 100%, where a higher value indicates better identification performance.

**Software tools and Configurations:** The selection of Regions of Interest (ROIs) and the calculation of mean spectra were performed in MATLAB R2018b. The correction and visualization of hyperspectral image data were carried out in the Environment for Visualizing Images (ENVI) 5.3 software from ITT Visual Information Solutions, Inc. in Boulder, CO, USA.

The experiments for the deep learning models proposed were conducted on a server equipped with an Intel Xeon Gold 5218 CPU (128 GB RAM) and an NVIDIA GTX 2080Ti graphics card (GPU), running in the Ubuntu Linux 21.04 operating system. The model’s compilation was created in the Python programming language (Python 3.7.10) and implemented using PyTorch 1.11.0 and CUDA 11.4. During the network training, the cross-entropy loss function and the Adam optimization algorithm were utilized, while the learning rate, dropout rate, and batch size were set to 0.0001, 0.3, and 16, respectively. The dataset was randomly split into a training set (90% of the dataset) and a test set (10% of the dataset). To ensure the reliability of the results, all of the experiments were conducted and averaged over 30 independent runs.

To evaluate the effectiveness of our developed algorithm, we conducted a comparative analysis with three widely recognized classic algorithms: Support Vector Machine (SVM), Multi-Layer Perceptron (MLP), and Random Forest (RF). SVM is a powerful supervised learning algorithm used for classification and regression tasks. It aims to find a hyperplane that best separates data points of different classes by maximizing the margin between them. In the SVM model, we utilized the Gaussian kernel function, known for its efficacy in non-linear classification tasks. MLP is a type of artificial neural network known for its capability to model complex relationships in data. It consists of multiple layers of interconnected nodes or neurons. For the MLP model, ReLU (Rectified Linear Unit) was employed as the activation function within the hidden layer. Additionally, we configured the model with a maximum of 200 iterations and an initial learning rate of 0.0001. Random Forest is an ensemble learning method that combines the outputs of multiple decision trees to make more accurate predictions. It is robust and can handle both classification and regression tasks. In the case of the RF model, we set the number of decision trees to 200, enhancing the model’s predictive power, and established a maximum depth of 40, ensuring a suitable trade-off between model complexity and performance.

### 3.2. Classification Results of FCB and Non-FCB

#### 3.2.1. Spectral Profiles of FCB and Non-FCB

**Figure 3:**
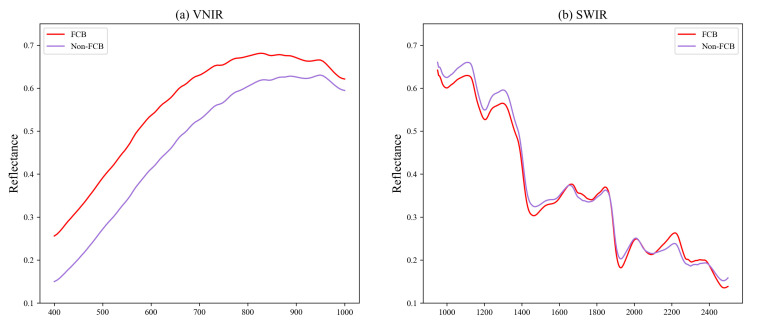
The average spectral reflectance of FCB and non-FCB.

With the ROI method introduced in Section 2.3, we calculated the mean spectral reflectance for FCB and non-FCB in all of the samples, and the results are shown in Figure 3. Panels (a) and (b) of Figure 3 correspond to the SWIR and VNIR lenses, respectively, covering a spectral range of 410.41–990.10 nm and 948.72–2512.97 nm. The mean spectral reflectance provides information on the basic characteristics of the samples.

It is evident that the spectral curve follows a similar trend overall, with peaks and valleys appearing in comparable positions. Nonetheless, there are noticeable differences in peak and valley values between the spectra. These differences can be attributed to variations in chemical composition and structure between the two types of samples. Therefore, the differences in spectral characteristics indicated that hyperspectral imaging had the potential to distinguish between FCB and non-FCB.

#### 3.2.2. Classification Performance Based on Machine Learning and an 1DCNN

At first, SVM, MLP, RF, and 1DCNN models were constructed without utilizing the attention mechanism. Table 3 displays the mean classification accuracy of the SVM, MLP, RF, and 1DCNN models for the two datasets, with optimal outcomes highlighted in bold. Based on the outcomes in Table 3, it is evident that the SVM, MLP, and RF models performed similarly and poorly, while the 1DCNN achieved the best performance across all datasets. Overall, the SWIR lens outperformed the VNIR lens. This can be explained by the spectral curve of the VNIR lens, as illustrated in Figure 3a, which displays less prominent differences in peak and valley values.

The poor performance of traditional algorithms can be attributed to the significant amount of redundant information present in hyperspectral data. The large amount of data and high correlation between characteristic bands makes sample identification challenging. Consequently, for traditional algorithms, the wavelength selection algorithm is usually necessary and required. Nevertheless, for an 1DCNN without a complex design, it was still higher than 90%. This also proves the superiority of the deep learning algorithm. That is, it can automatically learn lower-level features in a layer-by-layer manner, which then combine to form abstract higher-level attributes that enable the discovery of distributed representations of the data. In this case, the 1DCNN model overcomes this drawback by performing a feature extraction of spectral information through convolution and pooling layers. Still, there is room for improvement, particularly in the visible light range. In the following sections, we will demonstrate how attention mechanisms can provide significant performance improvement.

#### 3.2.3. Effectiveness of the CAM, SAM, and CSAM

**Figure 4:**
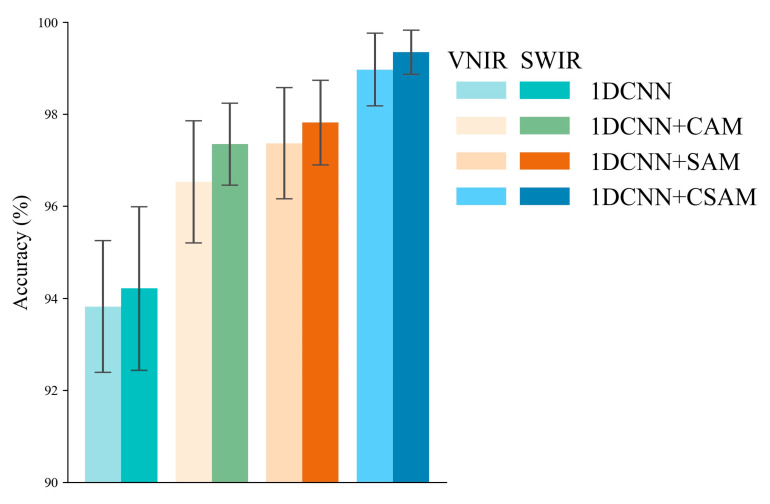
Classification results of the 1DCNN, CAM–1DCNN, SAM–1DCNN, and CSAM–1DCNN models established by FCB and non-FCB.

**Figure 5:**
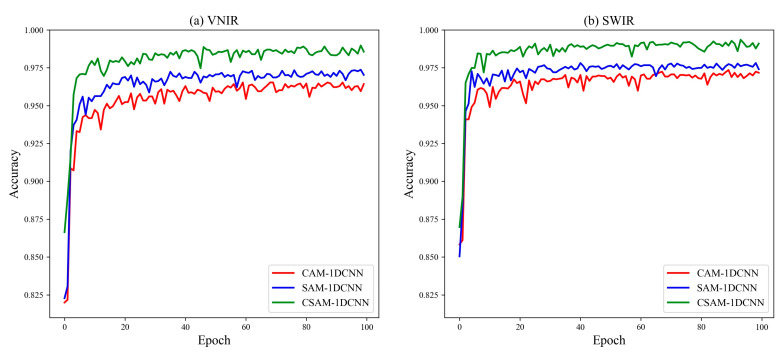
Performance comparison of the CAM–1DCNN, SAM–1DCNN, and CSAM–1DCNN established by FCB and non-FCB.

In this subsection, three attention mechanisms were utilized to elevate the classification accuracy based on the 1DCNN model. To investigate the effects of different modules in more detail, several different combinations of experiments were conducted. The final classification results are summarized in Figure 4, and the training accuracy of the three models for the first 100 epochs are illustrated in Figure 5.

Evidently, the attention mechanisms enhance the classification performance of the two lenses. Firstly, CAM–1DCNN specifically achieves an average accuracy increase of 2.71% and 3.14% for the two lenses, respectively, confirming the effectiveness of CAM in enhancing feature extraction ability and improving feature map selection. By comparison, SAM–CNN shows even higher performance gains, reaching accuracies of 97.37% and 97.82% for the two lenses. The reason is that SAM focuses more on the importance of different spectral bands, which is a key issue in hyperspectral data processing. Moreover, combining both mechanisms in CSAM–1DCNN produces the best performance, as expected, with accuracies of 98.97% and 99.35% for the two lenses.

In summary, the performance gain of introducing attention mechanisms is noticeable. These experimental results also provide strong evidence for the effectiveness of the proposed algorithm, which highlights that the introduction of attention mechanisms can better exploit hyperspectral feature information.

### 3.3. Classification Results of Fritillaria Commodity Specifications

To further validate the superior performance of the proposed model, we conducted a more detailed eight-class classification of the collected *Fritillaria* dataset.

#### 3.3.1. Spectral Profiles of *Fritillaria* Commodity Specifications

**Figure 6:**
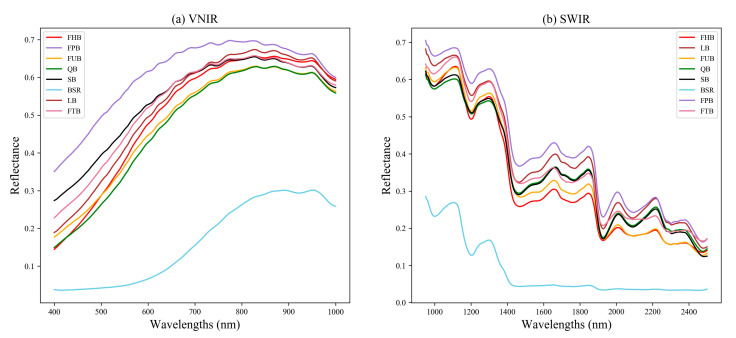
The average spectral reflectance of eight different species of *Fritillaria*.

Similarly, Figure 6 displays the average spectral reflectance for each species. All of the species, except for the BSR sample, exhibit similar waveform trends. However, the peaks and valleys, which are closely linked to species composition, show greater differences. These differences can be attributed to the fact that in Figure 3, the reflectance of both species was averaged. Notably, the spectral curve of the BSR species differs significantly from the others, as it does not belong to the family of Liliaceae. To summarize, the more notable differences among these spectra again underscore the potential of HIS as a viable option for identification purposes.

#### 3.3.2. Classification Performance Based on Classical Algorithms

We initially utilized three machine learning models, namely SVM, MLP, and RF, to classify different *Fritillaria* species, and Table 4 presents the classification accuracy of these models. However, no distinctive difference in classification performance was observed among these models, and the results were suboptimal, especially when using the VNIR lens. These outcomes further demonstrate the limitations of conventional algorithms, which heavily rely on band selection, particularly for more complex multi-class classification tasks, resulting in reduced performance.

#### 3.3.3. Effectiveness of the 1DCNN and Various Attention Mechanisms

**Figure 7:**
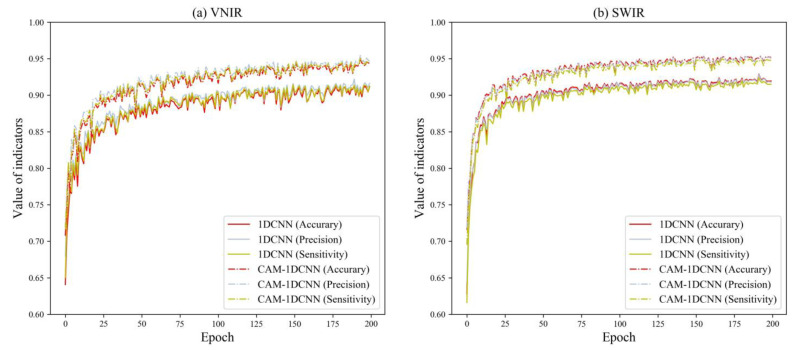
Performance comparison of the 1DCNN and the CAM–1DCNN models established by the samples of eight different species of *Fritillaria*.

**Figure 8:**
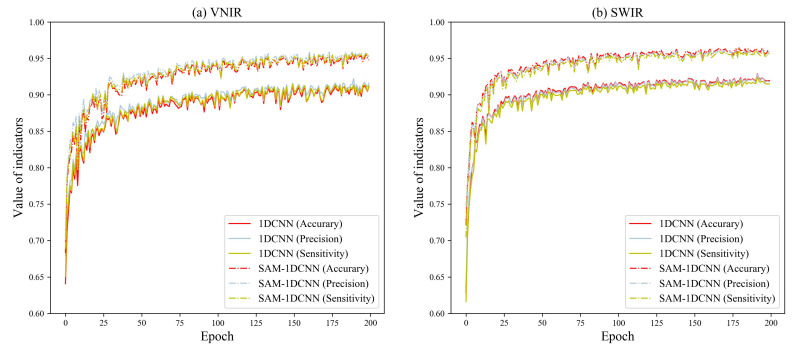
Performance comparison of the 1DCNN and the SAM–1DCNN models established by the samples of eight different species of *Fritillaria*.

**Figure 9:**
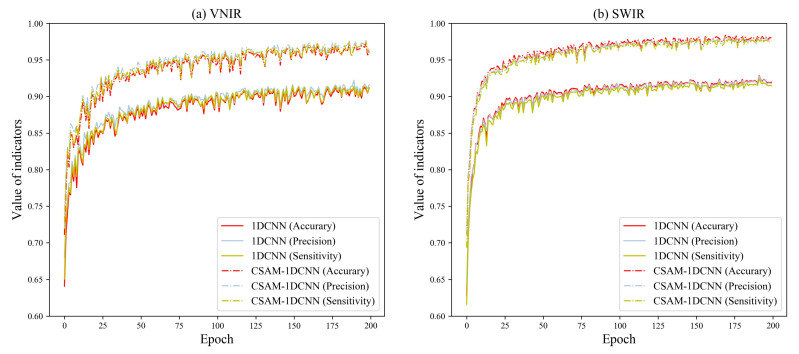
Performance comparison of the 1DCNN and the CSAM–1DCNN models established by the samples of eight different species of *Fritillaria*.

Table 5 displays the classification results of the 1DCNN, CAM–1DCNN, SAM–1DCNN, and CSAM–1DCNN modules. To facilitate a comprehensive analysis, we present the training curves of each network in Figure 7, Figure 8 and Figure 9. Specifically, Figure 7, Figure 8 and Figure 9 showcase their performance in terms of accuracy, precision, and sensitivity over the first 200 epochs. Overall, the outcomes indicate that, even for the more complex eight-class classification task, both the 1DCNN and its enhanced versions maintain excellent performance, surpassing 90%. This highlights a clear superiority in performance compared to traditional algorithms.

First, under the VNIR lens, the CAM–1DCNN model achieved an increase of 3.57%, 3.25%, and 3.67% in accuracy, precision, and sensitivity, respectively, in comparison to the 1DCNN model. Meanwhile, under the SWIR lens, the CAM–1DCNN model achieved an increase of 2.45%, 2.44%, and 2.49% in accuracy, precision, and sensitivity, respectively. This improvement can be credited to the CAM’s capacity to effectively select the most informative channels in the feature maps.

Secondly, the SAM–1DCNN module obtained better performance, as evidenced by the accuracy, precision, and sensitivity values on the VNIR lens, increasing by 4.21%, 3.59%, and 4.31%, respectively. Similarly, under the SWIR lens, the SAM–1DCNN achieved an increase of 3.57%, 3.38%, and 3.91% in accuracy, precision, and sensitivity, respectively. Again, these improvements were even more pronounced compared to the CAM–1DCNN module, highlighting the SAM’s ability to acquire highly correlated spectral features with *Fritillaria* characteristics while effectively filtering out irrelevant spectral information. These findings further reinforce the importance of spectral attention mechanisms.

Thirdly, when compared to the 1DCNN, the CSAM–1DCNN exhibited marked improvements of 5.97%, 5.44%, and 6.06% as well as 5.51%, 5.48%, and 5.91% in accuracy, precision, and sensitivity, respectively, under the VNIR and SWIR lenses. Simultaneously using the CAM and SAM achieved the best classification performance, with the accuracy, precision, and sensitivity values for all eight types of *Fritillaria* exceeding 97%.

To summarize, these results are consistent with those mentioned in Section 3.2.3 for the classification of different types of *Fritillaria*. Again, these findings reinforce the effectiveness of the attention mechanism, demonstrating its high robustness in accurately identifying various *Fritillaria* species.

**Figure 10:**
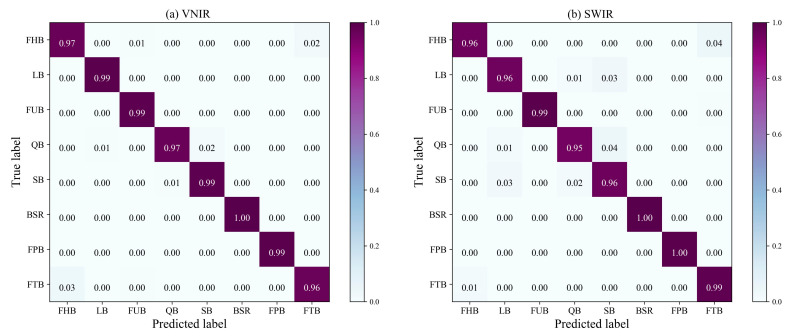
Confusion matrix of the CSAM–1DCNN.

Finally, to delve deeper into the performance of the best CSAM–1DCNN model, we present a detailed confusion matrix in Figure 10. In Figure 10, the columns and rows denote the true and predicted labels, respectively, and the diagonal elements correspond to the classification accuracy of the eight distinct *Fritillaria* species. As illustrated, over 95% of the *Fritillaria* species were identified properly, while less than 5% were misclassified. In particular, our findings indicate that the CSAM–1DCNN model achieves a classification accuracy of 100% under both lenses for BSR. This is supported by the distinctive BSR spectral curve displayed in Figure 6, which differs markedly from those of other *Fritillaria*. The confusion matrix provides additional evidence of the model’s efficacy in accurately distinguishing between different *Fritillaria* species, with high precision.

## 4. Discussion

The primary objective of our study was to enhance the accuracy and efficiency of *Fritillaria* species identification. By training our enhanced 1DCNN model with an attention mechanism on hyperspectral data, we achieved remarkable results. The model demonstrated the ability to distinguish between species, even when visual differences were subtle. This significantly contributes to the fields of botany and plant taxonomy, as it offers a non-invasive and highly accurate method for species differentiation.

The field of hyperspectral imaging has made significant strides in recent research, particularly when harnessed in combination with multivariate analysis and artificial intelligence. In the context of our study, which was focused on the identification of *Fritillaria* species using hyperspectral imaging and an enhanced 1DCNN with an attention mechanism, it is imperative to position our findings within the dynamic landscape of hyperspectral imaging applications. The work by Alimohammadi et al. [32] exemplifies the power of integrating spectral data and machine learning techniques for the classification of biological materials. This aligns seamlessly with our approach, where we harnessed spectral data to effectively differentiate between distinct *Fritillaria* species. Their study underscores the substantial potential of hyperspectral imaging for species classification, a facet that resonates with the outcomes of our research. Nalepa et al. [62] provide valuable insights into the contemporary challenges, trends, and opportunities within hyperspectral data analysis. Our study adds to this ongoing dialogue by introducing an innovative application of hyperspectral imaging in the domain of species identification. The exploration of spectral signatures within *Fritillaria* species serves as a testament to the adaptability and versatility of this technology, echoing the dynamic landscape discussed in this reference. Grosjean et al. [63] emphasize the non-invasive character of hyperspectral imaging, which seamlessly aligns with our approach, placing a premium on non-invasive species identification. Our work expands the horizons of hyperspectral imaging by demonstrating its applicability in the study of plant species, reflecting the innovative methodology expounded in this reference. Furthermore, Bauriegel et al. [64] illustrate the utility of hyperspectral imaging in exploring biological interactions. In a similar vein, our study leverages spectral signatures for the differentiation of *Fritillaria* species, underscoring the versatility of hyperspectral imaging in the study of biological systems. Both studies collectively affirm the potential of hyperspectral imaging for biological and botanical investigations.

In a word, our research contributes to the ever-evolving field of hyperspectral imaging applications. The combination of advanced deep learning techniques and attention mechanisms enables precise and non-invasive *Fritillaria* species identification. As hyperspectral imaging continues to advance, we anticipate further breakthroughs and applications across various scientific disciplines.

## 5. Conclusions

In this study, we developed an efficient and non-destructive detection method based on an 1DCNN for identifying different *Fritillaria* species. To alleviate the issue of redundancy and collinearity in high dimensional hyperspectral data, we innovatively introduced two novel attention mechanisms and combined them both to enhance vanilla 1DCNN network performance. With this strategy, the proposed enhanced 1DCNN with an attention mechanism can adaptively optimize the features of the input spectral information and encode the most critical features. The experimental results exhibit the superior capability of the proposed method to achieve high accuracy and reliability, particularly in the context of complex eight-category classification tasks involving *Fritillaria* species.

To the best of our knowledge, this is the first time that deep learning has been combined with an HIS technique for *Fritillaria* identification. Overall, the results illustrated the great feasibility of the authentication of *Fritillaria* species with the combination of hyperspectral imaging and a deep learning method. Moreover, it is important to note that the success of the attention mechanism modules in enhancing the performance of the 1DCNN model is worthy of further exploration. Similarly, for the quality control of the plants usable for both pharmaceutical and alimentary purposes, the attention mechanism modules can be effectively applied and can be expected to consolidate the performance of existing deep learning networks.

## Figures and Tables

**Table 1:** The numbers of samples of *Fritillaria*.

Lenses	Other *Fritillaria* Except FCB					Fritillariae Cirrhosa Bulbus		
	FTB	FPB	FUB	FHB	BMR	SongBei	QingBei	LuBei
VNIR	4041	4344	4497	3919	4696	6355	4556	6423
SWIR	4105	4317	4399	3887	4571	6235	4271	5643

**Table 2:** The network configuration of the CSAM–1DCNN architecture under the SWIR lens.

Type	Kernel	Channel	Steide	Padding	Output
Input					1 × 288
BN1					1 × 288
Conv-1/pooling	9 *×* 1	16	1	Yes	16 × 144
CPA	1 *×* 2	1	1	No	16 × 144
BN2					16 × 144
Conv-2/pooling	7 *×* 16	16	1	Yes	16 × 72
CPA	1 *×* 2	1	1	No	16 × 72
BN3					16 × 72
Conv-3/pooling	5 *×* 16	32	1	Yes	32 × 36
CPA	1 *×* 2	1	1	No	32 × 36
BN4					32 × 36
Conv-4/pooling	3 *×* 32	64	1	Yes	64 × 18
CPA	1 *×* 2	1	1	No	64 × 18
BN5					64 × 18
Conv-5/pooling	3 *×* 64	64	1	Yes	64 × 9
BN6					64 × 9
Conv-6/pooling	3 *×* 64	32	1	Yes	32 × 4
Fc1					64
Fc2					32
Fc3					16
Fc4					8

**Table 3:** Classification results obtained by the SVM, MLP, RF and 1CDNN models.

Models	VNIR Lens	SWIR Lens
SVM	85.40 ± 0.63	88.42 ± 0.78
MLP	84.96 ± 1.02	87.29 ± 1.41
RF	85.39 ± 0.89	86.80 ± 0.77
1DCNN	93.82 ± 1.44	94.21 ± 1.78

**Table 4:** Classification results obtained by the SVM, MLP, and RF models.

Models	VNIR Lens	SWIR Lens
SVM	44.15 ± 2.36	51.39 ± 1.98
MLP	46.73 ± 1.49	53.29 ± 1.69
RF	44.73 ± 0.88	54.92 ± 1.02

**Table 5:** Classification results of the 1DCNN, CAM–1DCNN, SAM–1DCNN, and CSAM–1DCNN modules.

Lenses		1DCNN	1DCNN+CAM	1DCNN+PAM	1DCNN+CPAM
VNIR	Accuracy	92.88	95.33	96.45	98.39
	Precision	92.80	95.24	96.18	98.28
	Sensitivity	92.40	94.89	96.31	98.31
SWIR	Accuracy	91.31	94.88	95.52	97.28
	Precision	92.20	95.45	95.79	97.64
	Sensitivity	91.39	95.06	95.70	97.45

## Data Availability

The data used to support the findings of this study can be made available by the corresponding author upon request.
